# Unravelling spermatogenesis in spotted wolffish: Insights from the ultrastructure of juvenile male testes to the cryopreservation of broodstock sperm

**DOI:** 10.1016/j.aquaculture.2024.741214

**Published:** 2024-11-15

**Authors:** Joshua Superio, Julien Resseguier, Rafael Henrique Nobrega, Caroline M. Grebstad, Ioannis Fakriadis, Atle Foss, Ørjan Hagen, Meiling Zhang, Maria del Pilar García-Hernández, Jorge Galindo-Villegas

**Affiliations:** a Department of Genomics, Faculty of Biosciences and Aquaculture, Nord University, Bodø 8049, Norway; b Section for Physiology and Cell Biology, Departments of Biosciences and Immunology, University of Oslo, Oslo, Norway; c Reproductive and Molecular Biology Group, Department of Structural and Functional Biology, Institute of Biosciences, São Paulo State University (UNESP), 18618-970 Botucatu, São Paulo, Brazil; d Institute of Marine Biology, Biotechnology and Aquaculture. Hellenic Center for Marine Research, Heraklion, Greece; e Akvaplan-Niva, Fram Centre, 9296 Tromsø, Norway; f Department of Aquaculture, Faculty of Biosciences and Aquaculture, Nord University, Bodø 8049, Norway; g Laboratory of Aquaculture Nutrition and Environmental Health (LANEH), School of Life Sciences, East China Normal University, Shanghai 200241, China; h Department of Cell Biology and Histology, Faculty of Biology, University of Murcia, Campus de Espinardo, 30100 Murcia, Spain

**Keywords:** *Anarhichas minor*, CASA, Cryopreservation, Emerging teleost species, Extenders, Phalloidin, Spermatogenesis

## Abstract

The aim of this study was to deepen our understanding of the reproductive biology of male spotted wolffish (*Anarhichas minor*) using two different experimental approaches involving juvenile and mature broodstock fish. The first approach consisted of a detailed histological examination of the testes to identify the onset of gonadal maturation and characterise the spermatogenic stages in two- and three-year-old juvenile specimens. Light microscopy analysis revealed clear differences between the age groups. Two-year-old fish displayed well-defined interstitial tissue, Sertoli cells and cysts housing spermatogonia stem cells in which meiosis had not yet begun. In contrast, three-year-old fish exhibited cysts containing spermatocytes, spermatids and abundant spermatozoa, indicating the initiation of the spermatogenic cycle, albeit with asynchronous puberty. Histochemical staining revealed a significant presence of smooth myoid cells in the interstitial tissue of sexually mature fish, while electron microscopy further revealed synaptonemal complexes indicating the onset of meiosis and centriolar structures that gave rise to flagella. The second approach focused on optimising semen freezing and cryopreservation procedures in mature broodstock individuals over the age of 10 years. Seven freezing extenders (KT, TS-2, OP, MT, MH, HBSS, or SR), with seawater (SW) as a control, were assessed along with two cryoprotectants dimethylsulfoxide (DMSO) or methanol to evaluate their impact on pre- and post-thaw semen quality. Results showed that the MT and HBSS extenders were superior in total sperm kinetics at 1:3 dilution, and that DMSO showed optimal results in sperm motility and velocity variants. Moreover, the MT and HBSS groups demonstrated consistent sperm viability after cryopreservation, with values similar to fresh samples. Based on the viability results of the SYBR-green-14/PI assay comparing fresh and cryopreserved sperm using MT and HBSS, the MT extender emerged as the most effective freezing medium for cryopreservation of spotted wolffish broodstock sperm. In conclusion, this study provides a comprehensive understanding of the reproductive dynamics of male spotted wolffish, offering valuable insights for both scientific research and aquaculture management.

## Highlights

• At the age of two years, a spotted wolffish weighs 2 kg on average, but is still sexually immature.
• At three years, 90% of the individuals sampled show complete spermatogenesis.
• The ultrastructural analysis additionally unveils evidence of asynchronous puberty.
• For adult broodstock sperm, the optimal extender is MT (1:3 ratio) and 10% DMSO.
• The reproductive capacity of males of this species appears viable in young fish.

## 1 Introduction

Gametes are the fundamental transferable units that carry genetic variability and information. They are also crucial for driving the evolution of populations and ensuring the survival and management of the species. Studies on the morphology of the gonads of teleost fish, particularly the male testes, have been pivotal in understanding various aspects of their reproductive biology (Parenti and Grier, 2004). Specifically, gross anatomical and histological studies have proven valuable in elucidating annual reproductive cycles (Hliwa et al., 2017), breeding seasons (Ribeiro et al., 2017), reproductive maturity (Dos Santos et al., 2019), spawning rhythms (Cowan et al., 2017), fecundity rates (Örği and Oğuz, 2022) and other critical aspects across various teleost fish species in aquaculture environments. At a more detailed level, histological ultrastructure of the male testes facilitates the characterisation of different germ cell types, allowing the identification of key reproductive events such as germ cell development, spermatozoa formation and tissue organisation (Schulz et al., 2010). However, puberty marks the onset of reproductive capacity in immature vertebrate animals for the first time. In teleost fish, puberty typically occurs after gonadal sex differentiation and is characterised by the initiation of meiosis in males and vitellogenic ovarian development in females. In aquaculture, early puberty can have negative effects on some species in terms of growth, feed utilisation, welfare and genetic effects of escapees in wild stocks (Florescu Gune et al., 2019; McClure et al., 2007; Taranger et al., 2010). Meanwhile, delayed or failed puberty prevents completion of the life cycle in captivity (van Ginneken et al., 2007). Consequently, this delayed puberty may result in extended culture time before maturation, which is not cost-effective in fish farming (Dufour et al., 2003). Therefore, understanding the mechanisms and timing of the onset of puberty is important in the aquaculture industry (Taranger et al., 2010) for improved management, especially in broodstock selection.

In general, many fish species show substantial sexual dimorphism during the reproductive season (Mei and Gui, 2015). For example, in captive spotted wolffish, assessment of the maturity stage of female fish is relatively straightforward, as the swelling of the belly can be tracked visually. Nevertheless, assessing the maturity of male fish sperm presents significant challenges, since they lack any easily observable external signs of maturity and do not display normal spawning behaviour (Kime and Tveiten, 2002; Le François et al., 2021a). Furthermore, indentifying males of quality is ideal for *in vitro* fertilisation, and requires scrutinising sperm characteristics (Kumaresan et al., 2020), with sperm motility and velocity being crucial factors (Fernández-López et al., 2022). To achieve quantitative and objective assessments of sperm motility, Computer-Assisted Sperm Analysis (CASA) systems have become indispensable tools (Kime et al., 2001; Mortimer and Mortimer, 2013). These systems use algorithms to quantify various parameters in individual sperm cells, generating extensive datasets on sperm motility properties. It should be noted that in most teleost fish species, whether marine or freshwater, spermatozoa remain quiescent in the testes and spermatic duct until they are released into the aqueous environment, where their motility is induced (Dietrich et al., 2016). However, unlike the sperm of most teleosts, the sperm of some species in particular, such as those of the *Anarhichadid* family, are usually extremely limited in quantity and, surprisingly, become motile upon stripping, remaining active for several hours without the need for activation (Kime and Tveiten, 2002). Cryopreservation is therefore recommended to facilitate reproduction in these specific species in confinement and to ensure a steady supply of sperm for synchronised gametes and genetic improvement programs (Alevra et al., 2022). However, this preservation technique may lead to decreased sperm quality and viability. This is particularly evident when the freezing medium extender fails to meet the desired standards, when cryoprotectants are inadequately chosen and included, and when the cryopreservation protocol, cryopreservation in liquid nitrogen and the subsequent thawing process lack precision. Despite the efforts made, achieving successful sperm cryopreservation remains a longstanding challenge, making it an elusive goal (Di Iorio et al., 2019).

Continuing advancements in aquaculture technology have enabled the farming of new species, providing alternative marketing avenues. These developments are instrumental in addressing the growing demand for edible protein while maintaining sustainability. However, despite the potential of numerous promising species to expand the fish farming industry, only a selected few have successfully emerged and become established (Acosta et al., 2024; Naylor et al., 2021). Indeed, the bottom-dwelling species of the *Anarhichadid* family, the spotted wolffish (*Anarhichas minor*), is a notable candidate for aquaculture expansion. Its suitability for aquaculture is further bolstered by its characteristics, such as rapid growth rates, efficient feed conversion and high-quality meat. It is found in the cold waters of Norway, spanning across the Atlantic and the Barents Sea, and its natural habitat is well suited to the conditions required for aquaculture (Foss et al., 2004; Superio et al., 2023), and its easy management in captivity makes it particularly attractive for aquaculture ventures (Le François et al., 2021b). However, understanding the captive biology of the spotted wolffish remains elusive. Generaly speaking, challenges in deciphering immune responses (Figueras et al., 2023), reproductive control (Fernandes da Costa et al., 2024), effective broodstock management (Che-Zulkifli et al., 2023) and improving general husbandry practices hinder the development of fish species for aquaculture. For example, in terms of reproductive biology, comprehensive studies on gonadal sex differentiation and the onset of spermatogenesis in juvenile spotted wolffish are lacking, althought knowledge has been gained on the ultrasturcutre of sperm in the closely related Atlantic wolffish (*A. lupus*) (Pavlov et al., 1997). Moreover, sexual maturity has been empirically stablished around 7–8 years (70–90 cm) for females and 8–9 years (80–85 cm) for males (Le François et al., 2021a), but no information is available regarding age and size at onset of puberty for this species. Furthermore, the availability of functional spermiating males has been limited, posing challenges for seedstock production at the hatchery level. It has also been suggested that the sperm of this species posess unusual characterisitcs in its trajectory and motility due to a spawning strategy that involves naturaly mixing the sperm with eggs in a gelatinous mass, rather than being released directly into the seawater water in proximity to the ova (Kime and Tveiten, 2002). Consequently, sperm cryopreservation protocols exist that can be used for artificial reproduction of the spotted wolffish (González-López et al., 2021; Santana et al., 2020). However, there is still much room for improvement in these procedures.

In this study, we have examined spermatogenesis in juvenile spotted wolffish using histological, histochemical and ultrastructural analyses. Moreover, we have expanded the existing research on sperm management (cryopreservation) of fully mature adult broodstock. The first experimental design of this comprehensive approach has allowed us to unravel intricate details of the reproductive biology of the spotted wolffish, offering a more thorough understanding of its spermatogenic development. In the second experimental design, we used fully functional sexually mature males to introduce new freezing extender media, assess the toxicity of two cryoprotectants and comprehensively examine available cryopreservation procedures. Overall, our study has provided valuable insights on spermatogenic processes and their functional management, significantly advancing our understanding of the reproductive biology of the spotted wolffish in captivity and at different stages of development.

## 2 Materials and methods

### 2.1 Ethical statement

All procedures were conducted with the approval of the institutional Animal Welfare Body, adhering strictly to the guidelines set forth in the Norwegian Animal Welfare Act (LOV-2009-06-19-97) and the European Union Directive (EU/2010/63) regarding the use of animals in research. Furthermore, explicit permission for the study was secured from the Norwegian Food Safety Authority (FOTS 28744).

### 2.2 Fish management and sample collection

**Fig. 1:**
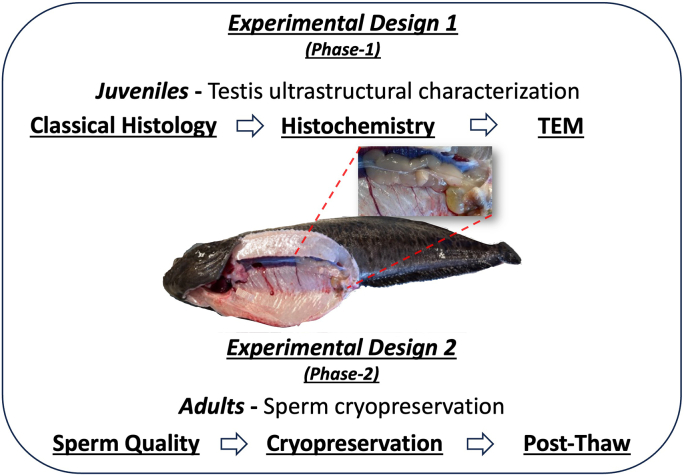
Experimental Setup – Two Phases. A comprehensive overview of an integrative study involving two distinct experimental phases conducted on spotted wolffish (*Anarhichas minor*), (Note the small size of the testes). In the first experimental design (*Phase-1*), tissue samples from testes of 2 to 3-year-old juvenile male specimens (*n* = 20 each cohort) were collected, fixed and subjected to ultrastructural analyses using classical histology, histochemistry (HC) and transmission electron microscopy (TEM). The second experimental design *Phase-2*) involved 40 broodstock males of mixed ages (10–12 years old). Sperm samples were collected and used to screen various extenders in two different sperm-to-extender ratios using two cryoprotectants under refrigeration and freezing. Sperm quality parameters were analysed using a Computer-Assisted Sperm Analysis (CASA) system.

To conduct this integrative study, two independent experimental designs were carried out with spotted wolffish (*A. minor*), across three developmental ages and stages of reproductive maturation. For clarity, a comprehensive summary detailing essential aspects of both experimental designs and the small size of the male testes is presented (Fig. 1). Notably, both juvenile (*n* = 300) and broodstock (*n* = 40) fish were continuously housed in separate 1600 L outdoor rectangular tanks at the Mørkvedbukta research station, Nord University, Norway (67° 16′ 41” N; 14° 33″ 25″ E). Each tank featured an open seawater flow-through system that maintained natural temperature (4–10 °C) and photoperiod conditions. Oxygen levels in the outgoing water of all tanks were maintained above 80% at all times. All fish were fed a commercial marine fish diet (Amber Neptun, Skretting, Norway), with 5 mm pellets for juveniles and 9 mm pellets for broodstock, administered *ad libitum* three times a day.

#### 2.2.1 Experimental design 1 (Phase-1)

Trial 1 was conducted in two stages. The initial batch of 20 two-year-old sexually immature fish, with an average body weight (BW) of 1.6 ± 0.1 kg and total length (TL) of 45.7 ± 1.1 cm, was sampled in early November 2021. The second batch, sampled in late October 2022, comprised of twenty naïve three-year-old male specimens from the same cohort as the previous year's fish (average BW of 3.3 ± 0.3 kg and TL of 55.1 ± 1.8 cm). On the day of sampling, animals were randomly collected directly from the fish stock and immediately immersed in a saturated solution of tricaine methanesulfonate (MS-222, Sigma-Aldrich) until death. Subsequently, to avoid excessive erythrocyte contamination of the target tissue, blood was collected from the caudal vein using 25-G needles attached to a 10 ml syringe (Castejón et al., 2021). After bleeding, each animal was dissected ventrally and its sex was visually confirmed. The testes were then collected following standard aseptic procedures. Each sample was then immediately divided into three identical pieces with a scalpel. Subsequently, each of the three pieces was immersed in Bouin's solution, 4% buffered formaldehyde and 2% paraformaldehyde/glutaraldehyde/cacodylate, respectively, for further ultrastructural analyses as detailed in section 2.3 below.

#### 2.2.2 Experimental design 2 (Phase-2)

Trial 2 was conducted during the reproductive season of the spotted wolffish, from November to February. The broodstock involved in this experiment consisted of 40 males of mixed ages (10–12 years old), with an average body weight (BW) of 10.3 ± 0.2 kg and a total length (TL) of 86.8 ± 0.5 cm. In each round of the experiment, a subgroup of five button-tagged fish was selected at two-week intervals until sperm was successfully obtained from each fish, forming a single batch. All fish in the batch were manually stripped on the same day. To facilitate the process, the fish were initially immersed in rectangular buckets filled with 100 L of seawater, containing a pre-optimised dose of 150 ppm of MS-222. This immersion lasted for five minutes until lack of movement indicated successful general anesthesia. Subsequently, the fish were gently placed on their sides and a gentle abdominal massage was administered to facilitate the release of feces and urine, thus minimising the risk of sample contamination. After eliminating biological waste, the urogenital pore was meticulously dried with a clean tissue. Subsequently, gentle pressure was applied to the lateral abdominal region containing the testes and sperm ducts, directing it towards the opening. Sperm samples were collected using a plastic Pasteur pipette with a cut tip, attached to the urogenital opening by suction, following a method previously described by (Superio et al., 2023). Collected sperm samples (*n* = 5) were kept at 4 °C and analysed within 1–3 h of initial collection. Only sperm samples collected with motility of 60% or higher were selected and used for the study, taking into account the number of fish that can be used for replicates.

### 2.3 Structural studies

#### 2.3.1 Classical histology

The testes obtained from 20 fish were fixed in Bouin's solution for 24 h, washed with 70% ethanol, embedded in Paraplast Plus (Sherwood Medical, Athy, Ireland) and sectioned at 5 μm thickness using a Leitz rotary microtome. After dewaxing and rehydration, specific sections were stained with hematoxylin–eosin to determine the reproductive stage of the specimens, following the protocol by (García Hernández et al., 2020). This staining procedure facilitated the analysis of serial sections of the testis. Bright-field histological images were captured using a Zeiss Axiolab microscope (Carl Zeiss, Germany) with the CoolSNAP image capture program (Roper Sci. Photometrics). A minimum of five sections of each specimen were analysed, ensuring a coefficient of variation of <10% between individuals.

#### 2.3.2 Histochemical staining (Phalloidin and DAPI)

After dissection, the testes were rapidly transferred to a 4% formaldehyde solution in HEPES buffer (60 mM, pH 7.4) and fixed for 24 h at room temperature, followed by a further 72 h at 4 °C, as outlined by (Resseguier et al., 2023). The fixed samples were then incubated twice in a solution containing 32% sucrose dissolved in distilled water for cryoprotection. After sinking to the bottom of the beaker, the samples were recovered, embedded in Tissue-Tek O.C.T. Compound (Sakura Finetek USA, Mountain View, CA, USA), flash-frozen in isopentane and sectioned using a CM1950 cryostat (Leica, Wetzlar, Germany). The resulting 30 μm cryosections were placed on Superfrost Plus slides (Thermo Fischer, Waltham, MA, USA) and stored at −20 °C. The slides were blocked for 1 h in a blockade solution (Thermo Fisher Scientific) at room temperature. Subsequently, the slides were co-stained with fluorescent phalloidin (TRITC or FITC labeled – Sigma Aldrich) at 3 U/mL and DAPI (Thermo Fischer, Waltham, MA, USA) at 5 μg/mL. Finally, the slides were mounted with prolong-glass mounting medium (Thermo Fischer, Waltham, MA, USA), cured at room temperature for 24 h, and stored at 4 °C prior to confocal imaging.

#### 2.3.3 Confocal image acquisition

Three-dimensional images were captured using the Zyla camera on a Dragonfly 500 spinning disk confocal microscope (Andor, Belfast, UK). The microscope was equipped with 40-μm pinholes and used either a 20×/0.75 dry objective or a 60×/1.4 oil-immersion objective. The acquisition, stitching and deconvolution processes (14 to 16 iterations) were carried out using the built-in features of the Fusion software. IMARIS and ImageJ/Fiji software were used for image analyses. The entire workflow, including image acquisition and analysis, were carried out on the NorMIC imaging platform at the University of Oslo, Norway.

#### 2.3.4 Transmission electron microscopy (TEM)

To prepare tissue specimens for TEM examination of the testicular structure of the spotted wolffish, the testes were finely chopped and fixed in a solution of 2% paraformaldehyde and 1% glutaraldehyde in 0.1 M cacodylate buffer (pH 7.2) at 4 °C for 2 h. After fixation, the samples were subjected to three washes in the same buffer solution and postfixed in a solution of 2% osmium tetroxide in 0.1 M cacodylate buffer (pH 7.2) for 1 h at 4 °C as described by (Dadras et al., 2023). Subsequently, the samples were dehydrated through a series of increasing concentrations of acetone (30%, 50%, 70%, 90%, 95%, and 100%) for 15 min each and embedded in resin (Epon 812, Germany). Ultrathin sections were then made using a Reichert-Jung ultramicrotome on copper grids, method according to the procedure by (van Bergen en Henegouwen and Leunissen, 1986). The sections were subjected to oxidation with sodium metaperiodate to restore specific labelling, followed by rinsing with distilled water and treatment with 50 mM PBS–glycine for 10 min. This was followed by three successive rinses with PBS containing 0.2% gelatin and 0.5% bovine serum albumin (PBG). Semithin sections used for pre-screening of the areas of interest at the histological facility were stained with toluidine blue. The ultrathin sections were contrasted with uranyl acetate and lead citrate and examined using Zeiss EM 10C and EM 109 electron microscopes. Sperm head diameter was determined using a MIP-Microm Image Processor (Microm España) based on the IMCO 10 system (Kontron Bildanalyse, Germany).

### 2.4 Sperm assessment

#### 2.4.1 Extenders preparation

In this study, seven extenders were prepared in-house and evaluated, together with seawater (SW). The extenders are as follows: Kime and Tveiten (KT) (Kime and Tveiten, 2002), TS-2 (Chen et al., 2004), Ocean pout (OP) (Yao et al., 2000), Modified turbot (MT) (Babiak et al., 2008), Modified halibut (MH) (Ding et al., 2011), Hank's balanced salt solution (HBSS) and Smith and Ryan (SR) (Smith and Ryan, 2010). In addition, seawater (SW) was directly obtained from the rearing environment and immediately filtered using a 0.22 μm pore size polyether sulfonate syringe filter (Sartorius, Germany). Detailed information on the exact composition, pH and osmolarity for each extender, SW and the spotted wolffish seminal plasma are presented (**Sup.** Table 1).

#### 2.4.2 Semen samples

Sperm collected as described in Section 2.2 (*Phase-2*) was subjected to several selection criteria prior to use. Specifically, only ejaculates that were clear whitish in colour, with no visual signs of urine or other contaminants, and with an estimated volume >0.5 mL were considered. Ejaculates meeting these criteria proceeded to the next stages, as described below.

#### 2.4.3 Sperm concentration

Sperm concentration was determined using a hemocytometer, specifically a Bürker cell-counting chamber. Briefly, the samples were vortexed and pre-diluted 100 times in different extenders according to the experimental stage. A 20 μL drop of the diluted sample was then placed in one of the hemocytometer chambers, which was mounted with a coverslip. The cells in the four diagonal squares within the large central square were counted using a microscope (Leica DM1000) equipped with a 40× lens. The total volume counted was 0.025 mm^3^. The results are expressed in cells per millilitre (cells ml^−1^).

#### 2.4.4 Sperm motility evaluation

Objective assessment of wolffish sperm was conducted using 5 μL of pre-diluted sperm with 1% bovine serum albumin (Sigma-Aldrich), which was placed in a CASA system (Computer Assisted Sperm Analysis) SCA 6.2–Motility module (Microptic, Barcelona, Spain). This system recorded several kinetic parameters, including total motility (%), curvilinear velocity (VCL), straight-line velocity (VSL) and average path velocity (VAP), measured in micrometres per second (μm/s). The CASA system was connected to a microscope (Nikon Eclipse Ci, Tokyo, Japan) equipped with a camera (Basler acA1300-200uc, Ahrensburg, Germany). To maintain consistency, the microscope stage temperature was regulated at 6 °C using a stage temperature controller (Linkam T95-PE, Tadworth, United Kingdom). The CASA system was adjusted to capture video at a rate of 20 frames per second, with a 1-s acquisition time and a head area of 10–50 μm^2^. Video recording began after any drift had ceased, typically between 1 and 2 min, and each sample was analysed in triplicate for accuracy and reliability.

#### 2.4.5 Sperm viability

Fish cell viability was assessed in freshly collected (*n* = 8) and post-thaw (*n* = 6) sperm samples utilizing fluorescence microscopy, following an established protocol (Cabrita et al., 2011). Briefly, 1 μL of sperm was diluted 1:100 in 1% NaCl, and a combination of 0.5 μL of propidium iodide (PI) (0.6 M) and 1 μL of SYBR-green-14 (resulting in a final concentration of 100 nM) dyes was applied to distinguish non-viable and viable cells, respectively. Each sample was subjected to microscopic examination under a fluorescent microscope (Nikon E200, Tokyo, Japan) with three images captured from different fields (magnified at 20×). Subsequently, a minimum of 100 cells per field were quantified using the “cell counter” feature within the ImageJ (Java) software. The results were expressed as percentage of viable cells.

### 2.5 Refrigeration, freezing and cryopreservation

#### 2.5.1 Screening of dilution ratio and different extenders in short-term preservation

After dilution and sperm mobility testing, the experimental batches mixed with the corresponding extender, in 1:1 or 1:3 dilutions, were placed in a household-type refrigerator at 2–4 °C. Periodic assessments of sperm motility were made at 72-h intervals to determine any differences in sperm motility and to establish the optimal dilution and storage period using the different extenders.

#### 2.5.2 Screening of two cryoprotectants, the top-five extenders and the optimal dilution under refrigeration

The study progressed by pairing the top five performing extenders at a dilution ratio of 1:3. The aim was to evaluate the toxic effect of adding 10% (*v*/v) of dimethyl sulfoxide (DMSO) or methanol (MeOH) as a cryoprotectant. Sperm integrity was assessed over a 24-h interval during refrigeration at 2–4 °C. This methodological approach aimed to discern the optimal combination to maximise sperm quality and survival during subsequent freezing procedures.

#### 2.5.3 Sperm cryopreservation and post-thaw quality assessment

The sperm samples were diluted at a ratio of 1:3 with the top five extenders (KT, OP, MT, HBSS and SR), each containing 10% DMSO as a cryoprotectant. After gentle mixing, the mixtures were stabilised on ice for 2 min before filling ten 0.5 ml cryo-straws per extender tested (Beirão and Ottesen, 2018). Subsequently, cryo-straws were frozen using freezing trays pre-filled with liquid nitrogen and containing a mesh suspended 4.5 cm above the limiting edge of the liquid nitrogen column for 10 min at a rate of −14.0 °C/min, as previously suggested (Santana et al., 2020). The frozen cryo-straws containing the sperm were then rapidly submerged in the liquid nitrogen and, after a short interval, recovered and placed in specific holders inside a dedicated cryopreservation device (Thermo, CK509X3, Bio-Cane 34). Moreover, the motility and viability of cryopreserved samples were compared using the top three extenders from the aforementioned set-up plus seawater as a negative control.

### 2.6 Statistical analysis

The data from the study were analysed using GraphPad Prism 10.0 software and presented as mean ± standard deviation (SD). One- or two-way ANOVA was used to analyse the difference between treatments. *P* values ≤0.05 were considered statistically significant.

## 3 Results

### 3.1 Structural studies – *Juvenile fish, (Phase-1)*

**Fig. 2:**
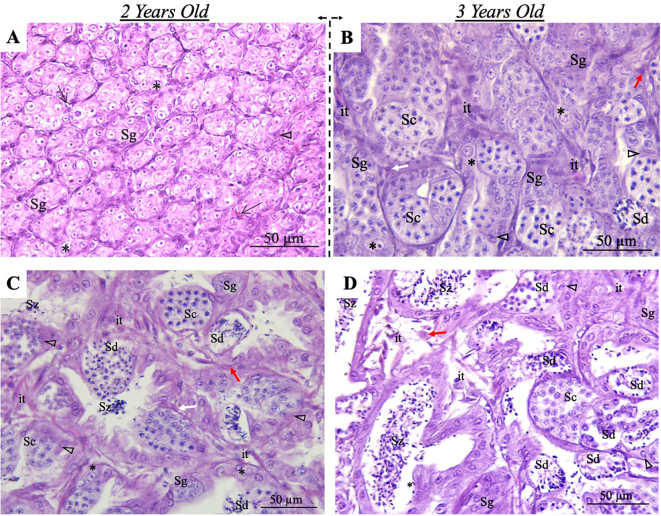
Cross section of the testis of spotted wolffish at two and three years of age. (A) Two-year-old male fish testes showing cysts containing type A undifferentiated spermatogonia (*) and spermatogonia (Sg). Arrows indicate interstitial tissue; open arrowheads show Sertoli cells. (B) In early spermatogenesis in three-year-old fish, the testis exhibits cysts containing type B spermatogonia (Sg), spermatocytes (Sc) and spermatids (Sd). The interstitial tissue (it) and Sertoli cell (open arrowhead) are also discernible. (C) Some males were found at more advanced stages of spermatogenesis, displaying cysts of spermatogonia (Sg), spermatocytes (Sc) and spermatids (Sd) but also with spermatozoa (Sz) within the lumen. (D) Males in late spermatogenesis were also observed, showing cysts of spermatogonia (Sg), spermatocytes (Sc) and spermatids (Sd), together with abundant spermatozoa (Sz) now present within the tubule lumen. Some immune cells such as acidophilic granulocytes and lymphocytes are also present (red and white arrows, respectively). Staining H&E. Bar = 50 μm. (For interpretation of the references to colour in this figure legend, the reader is referred to the web version of this article.)

In this study, we collected testicular samples from apparently healthy (by visual inspection) spotted wolffish individuals aged two and three years for structural characterisation. Initially, we focused on the testes of two-year-old males, revealing that they were at an immature stage in all individuals examined. In particular, a considerable number of cysts containing undifferentiated type A spermatogonia were observed, along with the presence of Sertoli cells, interstitial tissue potentially housing Leydig cells and various connective tissue elements (Fig. 2A). We then compared these findings with those obtained in testes of three-year-old fish. Our results indicated a significant change in maturity. Notably, the testes were in the initial spermatogenesis phase, showing type B spermatogonia and abundant cysts of spermatocytes, indicating spermatogonial differentiation and entry of meiosis (Fig. 2B). In addition, blood/lymph vessels, granulocytes and lymphocytes were identified among the distinguishable structures. Furthermore, some males (almost 90%) were found to be in a more advanced stage of spermatogenesis (spermiogenesis and spermiation), as indicated by the presence of spermatozoa within the lumen of the convoluted cluster of eferent ducts (Fig. 2C). We also reported males in the final stages of spermatogenesis exhibiting an abundant number of flagellated spermatozoa in the lumen, which is enlarged compared to earlier stages **(**Fig. 2D). Interestingly, we provided evidence for the first time that spotted wolffish at three years of age are already sexually mature, although they show asynchronous testicular development (precocious puberty).

**Fig. 3:**
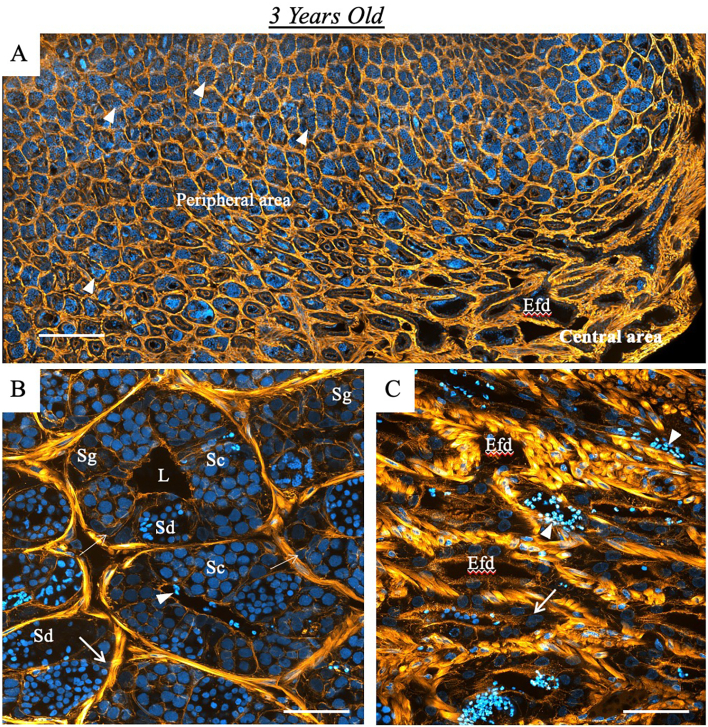
General organisation of the testis of the spotted wolffish. Representative deconvoluted confocal images of a 3-year-old juvenile spotted wolffish testis containing spermatozoa (indicated by arrowheads) as the most developed germ cell type, present in all tubules. (A) Lower magnification of a testis in the middle of spermatogenesis, with tubules showing germ cell cysts at various stages of spermatogenesis in the peripheral area, while the central area shows the efferent duct (Efd) system with a few spermatozoa in the duct lumen. (B) Provides a higher magnification of the peripheral area, showing the germinal compartment with different cysts of spermatogonia (Sg), spermatocytes (Sc) and spermatids (Sd). Additionally, Sertoli cells (arrows) are observed within the germinal compartment. (C) Higher magnification of the central area, highlighting the presence of peritubular myoid cells in the interstitial tissue (highlighted in yellow) surrounding the testicular duct. These images were obtained from 30 μm cryosections of testes stained with phalloidin (yellow) and DAPI (blue). (For interpretation of the references to colour in this figure legend, the reader is referred to the web version of this article.)

Next, we focused on the comprehensive architectural features of the testes in three-year-old fish. Using histochemical staining with phalloidin and DAPI, followed by confocal microscopy, we observed that peritubular myoid cells form a single cohesive layer surrounding the tubules. This investigation also revealed distinct structural domains within the testicular tubules. The peripheral region showed conspicuous clusters of germinative cells at various stages of development, providing clear evidence of the dynamic process of spermatogenesis. In contrast, the central area revealed the intricate network of testicular ducts, characterised by a reduced presence of spermatozoa within the duct lumen (Fig. 3A). Upon closer examination at higher magnification, the peripheral area displayed the classic cystic distribution of spermatogenesis, showing cysts of spermatogonia, spermatocytes and spermatids. We also observed Sertoli cells surrounding the germ cells (Fig. 3B), further elucidating the intricate microenvironment supporting spermatogenesis in the spotted wolffish. Finally, as shown in Fig. 3C, the central area of the tissue examined reveals a remarkable presence of peritubular myoid cells within the interstitial tissue. These cells are highlighted in yellow, showing their abundance and distribution. Amidst the interstitial milieu are the testicular ducts.

**Fig. 4:**
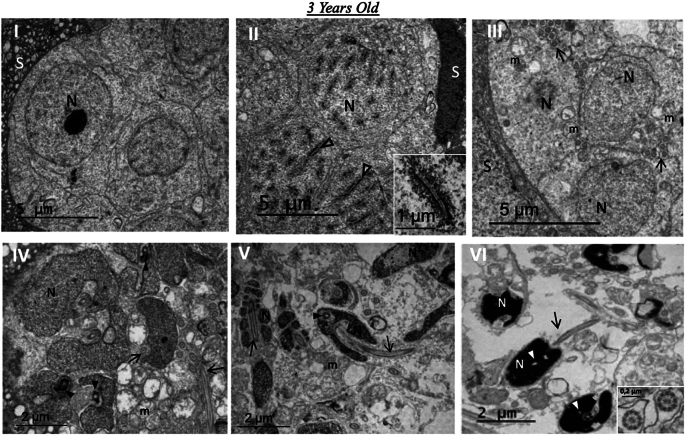
Spermatogenesis in 3-years old wolffish. (I-VI). Cysts of spermatogonia (I) and primary spermatocytes (II), surrounded by Sertoli cells (S). Notably, synaptonemal complexes indicative of prophase I of the first meiotic division are observed (open arrowheads, inset II). (III-VI). Differentiation of spermatids into spermatozoa (spermiogenesis). Note the progressive condensation of chromatin from initial spermatids (III) to spermatozoa (IV). In initial spermatids (III), the nucleus is round with euchromatic chromatin, surrounded by cytoplasm. As the spermatids differentiate, (IV-V), the nucleus becomes elongated and condensed. The centriolar complex, which gives rise to the flagellum, is located within a nuclear invagination (arrowhead in IV), which deepens as spermatids differentiate (arrowhead in V). The flagella have a 9 + 2 structure (inset in VI) regarded as axonemes. Spermatozoa have minimal cytoplasm and show uniformly condensed chromatin with small less condensed areas (white arrowheads in VI). Mitochondrion (m). Each micrograph, including the insets, provides a scale (1–5 μm) and scale bars. All micrographs were acquired by transmission electron microscopy (TEM).

Transmission electron microscopy (TEM) analysis revealed germ cells at different stages of spermatogenesis. Cysts of spermatogonia (Fig. 4 **I**) and primary spermatocytes (Fig. 4 **II**) were prominently observed and were surrounded by cytoplasmic extensions of Sertoli cells. Notably, the presence of synaptonemal complexes within primary spermatocytes (**inset 4 II**) confirmed the progression through prophase I of the first meiotic division, substantiating the ongoing cell division. Moreover, the subsequent stages of spermatid differentiation into spermatozoa were clearly delineated (Fig. 4 **III-VI**). Initial spermatids (Fig. 4 **III**) had a round nucleus containing euchromatic chromatin. As spermatids progressed in differentiation, the nucleus elongated and condensed (Fig. 4 **IV-V**), accompanied by a prominent nuclear invagination housing the centriolar complex, crucial for flagellum formation (**arrowheads in 4 IV-V**). Significantly, the flagella of spermatozoa showed a characteristic 9 + 2 axonemal structure (**inset 4 VI**). Additionally, the spermatozoa exhibited minimal cytoplasm and uniformly condensed chromatin, with small less condensed areas, consistent with the morphology of mature spermatozoa. Overall, the TEM analysis confirmed the above observations, providing a detailed view into the intricate process of spermatogenesis in 3-year-old spotted wolffish.

### 3.2 Sperm quality assessment and cryopreservation - adult fish, (Phase-2)

**Fig. 5:**
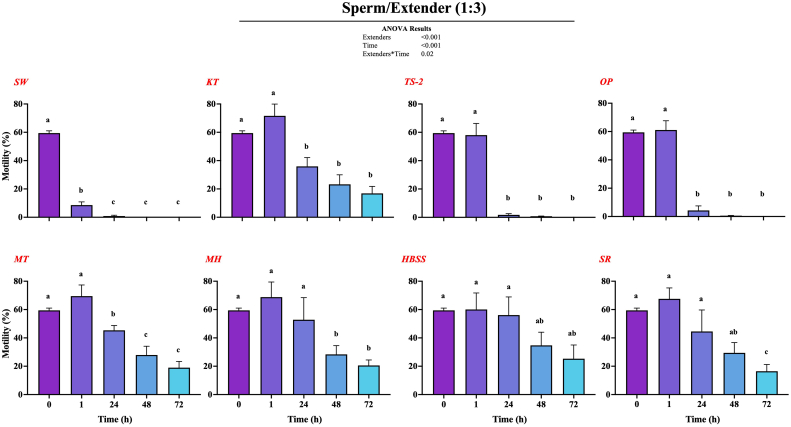
Effect of extender solutions on CASA parameters of diluted sperm from spotted wolffish broodstock (*n* = 3) over a 72-h period under refrigeration at 2–4 °C. Sperm diluted 1:3 with various extenders (SW, KT, TS-2, OP, MT, HBSS, or SR). The parameter evaluated was the motility rate (%). T0 represents the state of fresh sperm without the addition of extender. Statistical analysis was performed bye two-way ANOVA (Tukey's HSD, *P* ≤ 0.05).

In the second part of the study, we began with a detailed investigation to assess the influence of various extender solutions on the motility and viability of spotted wolffish broodstock sperm under chilled storage conditions (2–4 °C). Initially, we evaluated seven different extenders (KT, TS-2, OP, MT, MH, HBSS, and SR), along with a control group using SW, to determine their effectiveness at different sperm-to-extender ratios over 72 h under refrigeration. This comprehensive approach enabled us to thoroughly examine the impact of each extender on sperm motility in chilled environments. Throughout the experimental phases, we used a computer-assisted sperm analysis (CASA) system to evaluate sperm motility rate. All extenders, except SW used to dilute fresh sperm of the spotted wolffish broodstock at a 1:1 ratio (**Supp.** Fig. 1), showed increased sperm motility after 1 h in refrigeration. However, after 24 h, a significant decrease in motility was observed in all groups, except MH and SR, compared to the values recorded at 1 h. Subsequently, at 48 and 72 h, motility was substantially reduced, leading to a gradual depletion of sperm motility at the end of the trial period. Similarly, when sperm was diluted at a 1:3 ratio (Fig. 5), a similar pattern was observed as in the 1:1 dilution. However, at 1:3 dilution, among the tested extenders, MT, HBSS and SR demonstrated superior performance (*P* ≤ 0.05) in maintaining sperm motility at different time points. We observed more stable motility with minimal significant changes with MT and HBSS extenders. Furthermore, there was no significant difference between MT and HBSS at any time point (**Supp.** Fig. 2). Consequently, we selected the 1:3 dilution ratio for the subsequent phases of the experiment.

Next, to assess the impact of cryoprotectants on sperm quality, specifically dimethyl sulfoxide (DMSO) and methanol (MeOH), we performed a series of experiments using the top five extenders (KT, OP, MT, HBSS and SR) identified in our previous trial. These extenders were evaluated over 24 h of thawing after cryopreservation (**Supp.** Fig. 3). As anticipated, both DMSO (**Supp.** Fig. 3A) and methanol (**Supp.** Fig. 3B) induced a gradual decrease in motility, with an average decrease of 15% and 20%, respectively, for all extenders used. However, samples treated with DMSO as a cryoprotectant exhibited higher, more consistent and stable motility and velocity values (P ≤ 0.05) than those treated with methanol. Based on these results, DMSO was selected as the preferred cryoprotectant for subsequent phases of the study, due to its superior performance in preserving sperm quality and viability afer thawing.

**Fig. 6:**
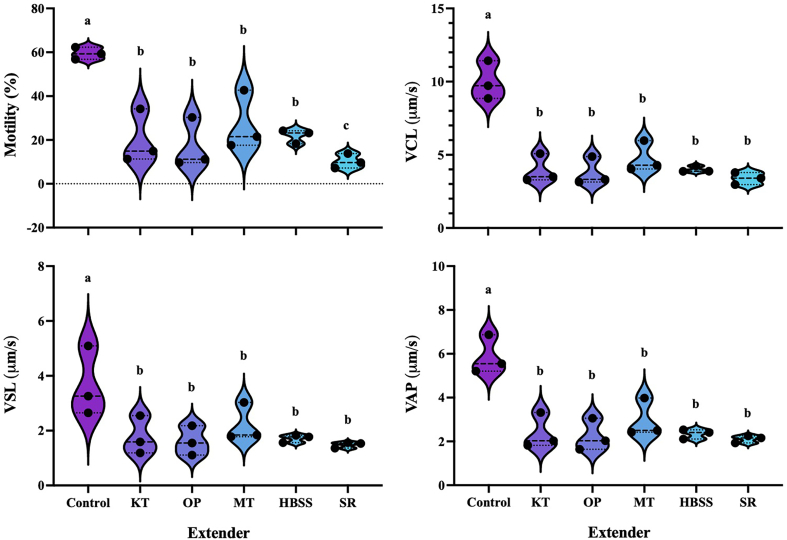
Impact of extenders combined with DMSO on the progressive motility and velocity variants of diluted sperm from broodstock spotted wolffish after 24 h of cryopreservation. Sperm diluted with various extenders (KT, OP, MT, HBSS or SR) in 10% DMSO and subsequently cryopreserved were subjected to analysis for motility rate (%), curvilinear velocity (VCL; μm/s), straight-line velocity (VSL; μm/s) and average path velocity (VAP; μm/s) after thawing 24 h after following cryopreservation and storage in a cryobank. The control represents the state of fresh sperm before the cryopreservation process. Statistical analysis was performed by one-way ANOVA (Tukey's HSD, *P* ≤ 0.05).

In the subsequent experiments, we aimed to determine the most effective extender based on sperm quality parameters after thawing. Sperm samples were diluted at a ratio of 1:3 (sperm to extender) using the top five extenders, each supplemented with 10% DMSO, and subjected to cryopreservation. After 24 h, the cryopreserved samples were thawed. Sperm motility parameters, including motility, curvilinear velocity (VCL), straight-line velocity (VSL) and average path velocity (VAP), were assessed using the computer-assisted sperm analysis (CASA) system (Fig. 6). The results revealed that all four extenders, as well as SW, showed significantly reduced motility and velocity compared to the same parameters assessed in fresh sperm without freezing. However, while MT produced the best results based on motility among KT, OP, MT and HBSS, no further changes were recorded in any of the sperm velocity parameters determined.

The three top extenders and seawater as an unformulated solution were then tested. The results revealed some variations in sperm concentration, quality, motility and number of live cells. However, for most parameters evaluated, no significant differences were observed between the two cryoprotectants tested, except for total motility, where DMSO showed a slightly higher performance. A comprehensive summary of the main characteristics essential for cryopreservation studies is presented (Table 1). The average values of the key markers of fresh sperm and of the cryoprotectants and extenders used for cryopreservation of adult broodstock of spotted wolfish sperm were evaluated. Of particular note is the comparison of fresh sperm with those subjected to samples containing 10% DMSO or methanol after cryopreservation. Interestingly, while for the SW the total motility is almost cero, at the same time the live cells percentage is still viable on the 75% level.

**Table 1:** Quantitative and qualitative indicators of fresh and cryopreserved sperm using two cryoprotectants and three extenders in adult broodstock wolffish sperm.

		Cryoprotectant		Extenders			
Average	Fresh	DMSO	MeOH	KT	MT	HBSS	SW
		(10%)	(10%)				
Volume (mL)	0,5-10,0	na	na	na	na	na	na
Sperm concentration (10^9^ ml^−1^)	1,5 ± 0,2 ^**A,a**^	0,6 ± 0,6^**B**^	0.5 ± 0,8^**B**^	0,5 ± 0,3^**b**^	0,6 ± 0,1^**b**^	0,4 ± 0,4^**c**^	0,4 ± 0,6^**c**^
Total motility (%)	62,2 ± 1,8 ^**A,a**^	40,6 ± 2,3^**B**^	36,2 ± 1,9^**C**^	23,2 ± 1,5^**b**^	33,0 ± 0,6^**c**^	16,6 ± 0,8^**d**^	2,0 ± 0,1^**e**^
VCL (μm/s)	10,0 ± 0,8 ^**A,a**^	4,1 ± 0,5^**B**^	3,7 ± 0,4^**B**^	4,0 ± 0,6^**b**^	4,8 ± 0,6^**b**^	4,0 ± 0,1^**b**^	1,1 ± 0,4^**c**^
VSL (μm/s)	3,7 ± 0,7 ^**A,a**^	1,6 ± 0,3^**B**^	1,4 ± 0,6^**B**^	1,8 ± 0,4^**b**^	2,2 ± 0,4^**b**^	1,7 ± 0,10^**b**^	0,8 ± 0,2^**c**^
VAP (μm/s)	5,9 ± 0,5 ^**A,a**^	2,2 ± 0,1^**B**^	1,8 ± 0,3^**B**^	2,4 ± 0,5^**b**^	3,0 ± 0,5^**b**^	2,4 ± 0,1^**b**^	1,2 ± 0,5^**c**^
Live cells (%)	95,7 ± 0,8^**a**^	nd	nd	91,3 ± 1,3^**a**^	93,7 ± 0,7^**a**^	87,5 ± 1,7^**b**^	75,1 ± 1,6^**c**^

The dilutions used for both cryoprotectant and extenders were a ratio of 1:3 sperm to extender. Different letters between the horizontal lines denote significant differences. Upper case letters represent comparisons between fresh sperm and cryoprotectant, while lower case letters represent comparisons between fresh sperm and KT, MT, HBSS extender, or seawater (SW). Curvilinear velocity (VCL), straight line velocity (VSL), average path velocity (VAP). Not applicable (na); No data (nd). Statistical analysis was performed by one-way ANOVA (Tukey's HSD, *P* ≤ 0.05).

## 4 Discussion

The present study represents a significant advance in providing valuable information on the reproductive biology and sperm management of spotted wolffish (*Anarhichas minor*), thus contributing to the fundamental knowledge required for successful aquaculture practices. Through a multifaceted and thorough experimental approach covering both juvenile and mature broodstock fish, we investigated various aspects of spermatogenic development, extender effectiveness, cryoprotectant impact and post-thaw sperm quality parameters.

Our research on the structural development of juvenile spotted wolffish has provided valuable insights into their spermatogenic progression. In two-year-old juveniles, signs of sexual maturity and asynchronous testicular development were evident. Features of puberty other than timing and age have generally been overlooked, although there is growing recognition that the variety of pubertal characteristics can deeply impact the reproductive status in fish (Taranger et al., 2010). These observations included well-defined interstitial tissue, the presence of Sertoli cells and cysts containing type A undifferentiated spermatogonia, which serves as the foundation of fish spermatogenesis (de Paiva Camargo et al., 2017). Conversely, our examination of three-year-old males revealed advanced spermatogenesis within their testes. This stage was characterised by the presence of cysts containing spermatocytes, spermatids and spermatozoa in the lumen, indicating the beginning of the spermatogenic cycle (Xie et al., 2020). Furthermore, building on our results and advancing towards precise terminology, the asynchronicity in the developmental stage of cells within the gonad is indicative of precocious puberty in this species. Previous studies have stablished that the age and size at puberty vary between and within species and strains, influenced by genetic and environmental factors, making it essential to understand these factors for optimising breeding practices and enhancing the sustainability of aquaculture (Taranger et al., 2010). The identification of pubertal age in spotted wolffish represents an important milestone in the aquaculture practice of this species. Puberty marks the transition from an immature juvenile to a sexually mature adult capable of reproduction (Taranger et al., 2010). With this discovery, the reproductive timeline of the spotted wolffish is entirely redefined, suggesting that males can enter their first reproductive stage much earlier than previously estimated, at approximately 7–9 years of age (Le François et al., 2021a). But in fact, the asynchronous testicular development observed in three-year-old fish underscores the complexity of reproductive maturation in this species, prompting further research to elucidate the underlying mechanisms. Furthermore, our results represent a breakthrough in the management of broodstock for this species, as no previous studies have established early sexual maturation in males. Establishing the onset of pubertal age for spotted wolffish males offers several avenues of manipulation that can further expand their aquaculture potential. For instance, since reproductive competence is acquired during puberty (Schulz et al., 2019), hormonal therapies could potentially increase the ability of this species to reproduce at an earlier age, eliminating the long waiting time to obtain broodstock previously suggested.

The results uncovered highlight a typical and well-defined pattern of spermatogenesis in the spotted wolffish, aligning with observations in numerous other fish species (Crespo et al., 2019; Hatef and Unniappan, 2019). In the spotted wolffish, this process unfolds in three distinct phases: the initial generation of spermatogonia by mitotic division, followed by the appearance of primary and secondary spermatocytes, and culminating in the emergence of motile haploid spermatozoa. These results are consistent with established knowledge in fish reproductive biology, where spermatogonia serve as the foundation of spermatogenesis (Cohen-Rothschild et al., 2024). Histochemical staining analyses further elucidated the testicular microenvironment in sexually mature spotted wolffish, revealing an abundance of peritubular myoid cells in the interstitial tissue. These cells typically form a single layer around the seminiferous tubules, providing structural and dynamic support to the tubules, in several fish species (Fernini et al., 2023). Additionally, our observations indicated the presence of leukocytes in close proximity to the developing spermatogonia, suggesting a possible critical immunoregulatory interaction between them and the germ cells, which are in direct contact with peritubular myoid cells, within the testicular environment. Pevious studies have shown that in seabream (*Sparus aurata*), testicular neutrophil-like cells constitutively produce interleukin-1β (*il1b*), which serves as a potent growth factor for spermatogonia and Leydig cells (Chaves-Pozo et al., 2005). In line with our findindings, we speculate that in the spotted wolffish, acidophilic granulocytes and posibly lymphocytes similarly influence the development and functionality of spermatocytes. However, further experimental validation is necessary to support this hypotesis, highlighting an intriguing avenue for future research into the immunological dynamics of the testis and sperm production at early developmental stages.

Next, our results expanded upon the ultrastructural characterisation of spermatogonia from juvenile male spotted wolffish, employing a multimodal approach that included TEM, complementing observations made using light and confocal microscopy techniques. Our findings revealed six distinct steps in the spermatogenic process of the spotted wolffish, delineated on the basis of the pattern and degree of chromatin condensation. While in some species, such as the European grayling, (*Thymallus thymallus*), this process is classified into more steps (Dadras et al., 2023). Interestingly, the micrographs obtained also revealed the presence of synaptonemal complexes and the emergence of the centriolar complex, shedding light on the intricate process of chromatin differentiation during spermatogenesis. The striking conservation observed in the spermatogenic cells of the spotted wolffish, as in other fish species, underscores the fundamental similarities in the underlying processes of fish spermatogenesis. However, the distinctive features observed in this species, such as the spatial arrangement of germ cells and the organisation of the interstitial compartment, emphasise the need for species-specific research to fully understand the complexities of reproductive biology in diverse taxa. To our knowledge, this is the first investigation using TEM to examine the ultrastructural characteristics of spermatogonia in the spotted wolffish. Previous research reporting the use of electron microscopy in wolffish has focused primarily on reproductive aspects of spermatozoa ultrastructure, but in a closely related species, the common wolffish (*Anarhichas lupus*) (Pavlov et al., 1997). Therefore, our findings contribute to a better understanding of cystic spermatogenesis in the spotted wolffish, offering valuable insights into the reproductive adaptations and complexities of this species. Furthermore, the results highlight the compelling need for continued research to elucidate the mechanisms underlying these observations and their potential implications for broader evolutionary and comparative reproductive biology.

As a continuation of our preceding histological analyses in juvenile fish, our research endeavors with adult broodstock have been directed towards the refinement of semen freezing and cryopreservation procedures, aimed at improving sperm quality and viability. While methodologies for such processes are well established in certain freshwater species, research concerning marine fish, including the spotted wolffish, have only recently gained attention (Asturiano et al., 2017). Our study, therefore, tried to assess seven different extenders paired with two cryoprotectants, with the primary objective of discerning the most effective combination for preserving semen quality before and after thawing. Our research has revealed the superior performance of the MT and HBSS extenders, especially when diluted at a ratio of 1:3, in maintaining overall sperm motility and viability. Of note is the predominant use of KT extender in previous protocols for cryopreservation of the spotted wolffish (Beirão and Ottesen, 2018; Santana et al., 2020), predominantly based on a formulation devised more than two decades ago (Kime and Tveiten, 2002). Additionally, a critical issue is the selection and subsequent application of cryoprotectants in the extender before performing a cryopreservation procedure. Usually, there is a balance between cryoprotectants efficiency and toxicity, that is variable between species and the choice of cryoprotectant (Zhang et al., 2005). Our experimental setting has unveiled the varying degrees of toxicity associated with the addition of cryoprotectants to these novel extenders, with DMSO exhibiting optimal outcomes concerning sperm motility and velocity. This observation aligns with previous investigations advocating DMSO in combination with KT as the most viable sperm cryoprotectant (Beirão and Ottesen, 2018). However, in the context of our present study, among the seven extenders scrutinised, MT, together with DMSO, has emerged as the most effective freezing medium for the cryopreservation of spotted wolffish broodstock semen, as corroborated by viability assays. Specifically, sperm samples stored with DMSO gave at least 4.5% higher motility than those stored with methanol, which means it is more protective and less toxic (Santana et al., 2020). Furthermore, the MT extender provided higher (∼10%) motility than the classic KT extender, which has been used for this species for decades (Kime and Tveiten, 2002; Santana et al., 2020). Ssignificant variations were also observed among SW and the three top extenders tested (KT, MT, HBSS), with SW resulting in the gradual arrest of sperm movement, highlighting the crucial role of osmolarity. Previous studies have determined that sperm motility is maximal at osmolalities between 200 and 500 mOsm (Kime and Tveiten, 2002). This range encompasses the osmolality of both freshwater and SW, and notably matches the 308 mOsm that we report (**Supp.** Table 1) for the seminal plasma of this species. These findings elucidate why this species does not release its sperm directly into the water during the spawning, as previously observed. Consequently, our results underscore the importance of selecting appropriate extenders to maintain sperm quality and viability during refrigeration or cryopreservation. Thius has practical implications for developing optimal sperm storage protocols, which are essential for the efective management of spotted wolffish broodstock. Furthermore, the results of this second part of the present study have significant implications for the aquaculture industry, particularly with regard to the management of spotted wolffish broodstock and the refinement of sperm cryopreservation. By elucidating the intricacies of the reproductive dynamics and sperm quality parameters of spotted wolffish, our study provides invaluable information for optimising breeding programmes, improving genetic diversity and promoting sustainable aquaculture practices.

## 5 Conclusion

Despite the progress made in this study, numerous avenues for future research need to be explored. Further investigation of the molecular mechanisms underlying spermatogenic development and puberty in the spotted wolffish is essential for a holistic understanding of reproductive maturation. In addition, continued refinement of sperm cryopreservation protocols, including the exploration of new extenders and cryoprotectants, is essential to optimise sperm quality and viability. Longitudinal studies evaluating the reproductive performance of cryopreserved sperm and the genetic integrity of the offspring are also warranted to determine the feasibility and efficacy of sperm cryopreservation in commercial aquaculture. In conclusion, this study offers a comprehensive evaluation of spermatogenesis and sperm management of spotted wolffish, providing valuable information to advance aquaculture practices and enhance sustainable production of this promising species. Further research efforts aimed at elucidating the intricacies of reproductive maturation and optimising sperm cryopreservation techniques at the molecular level will be critical to unlocking the full potential of spotted wolffish aquaculture.

## CRediT authorship contribution statement

**Joshua Superio:** Writing – original draft, Methodology, Investigation, Formal analysis, Data curation. **Julien Resseguier:** Methodology, Formal analysis, Data curation. **Rafael Henrique Nobrega:** Writing – original draft, Validation, Formal analysis. **Caroline M. Grebstad:** Methodology, Investigation. **Ioannis Fakriadis:** Methodology, Investigation. **Atle Foss:** Writing – original draft, Investigation. **Ørjan Hagen:** Resources. **Meiling Zhang:** Writing – original draft, Validation. **Maria del Pilar García-Hernández:** Resources, Methodology, Investigation, Formal analysis, Data curation, Conceptualization. **Jorge Galindo-Villegas:** Writing – review & editing, Visualization, Supervision, Resources, Project administration, Methodology, Funding acquisition, Formal analysis, Conceptualization.

## Declaration of competing interest

The authors declare the following financial interests/personal relationships which may be considered as potential competing interests.

Jorge Galindo-Villegas reports financial support was provided by 10.13039/501100000780European Commission, EU H2020 ERANET BlueBio Cofund. Jorge Galindo-Villegas reports financial support was provided by 10.13039/501100005416Research Council of Norway. Jorge Galindo-Villegas and Meiling Zhang declare that they are members of the editorial board at Aquaculture at the time of submission If there are other authors, they declare that they have no known competing financial interests or personal relationships that could have appeared to influence the work reported in this paper.

## Data Availability

Data will be made available on request.

## Appendix A Supplementary data

Supplementary material: Supplemental Table 1. Extenders composition. Image 1

Supplementary material: Supplemental Figure 1. Sperm/extender (1:1). Image 1

Supplementary material: Supplemental Figure 2. Impact of MT and HBSS. Image 1

Supplementary material: Supplemental Figure 3. Impact of cryoprotectants. Image 1
