# Supplementary material for: Unravelling spermatogenesis in spotted wolffish: Insights from the ultrastructure of juvenile male testes to the cryopreservation of broodstock sperm
Source: Aquaculture. 2024 Nov 15;592:741214. doi: 10.1016/j.aquaculture.2024.741214 (PMC11336258; doi:10.1016/j.aquaculture.2024.741214)
Supplement: Supplementary file 1 — Supplementary material: Supplemental Table 1. Extenders composition. [file mmc1.docx]

**Supplemental Table 1**

**Supplemental Table 1. Formulas of extenders, pH and osmolarity, together with physical parameters of seawater and spotted wolffish seminal plasma.**

| **Component** | **Units** | **Extenders** | | | | | | | **SW** | **Seminal Plasma** |
| --- | --- | --- | --- | --- | --- | --- | --- | --- | --- | --- |
|  |  | **KT** | **TS-2** | **OP** | **MT** | **MH** | **HBSS** | **SR** |  |  |
| NaCl | *mM* | 145 |  | 183 | 70 |  | 138 | 207 |  |  |
| KCl |  |  |  |  | 1.5 |  | 5.3 | 5.4 |  |  |
| CaCl_2_ |  | 4.55 |  | 1.45 | 2.7 |  | 1.26 | 1.3 |  |  |
| MgCl_2_ |  |  |  |  | 6.1 |  | 0.5 | 0.49 |  |  |
| MgSO_4_ |  | 2.37 |  | 0.84 |  |  | 0.41 | 0.41 |  |  |
| KHCO_3_ |  | 4.83 | 100 | 10.25 |  |  |  |  |  |  |
| KH_2_PO_4_ |  |  |  |  |  |  | 0.44 |  |  |  |
| Na_2_HPO_4_ |  |  |  |  |  |  | 0.34 |  |  |  |
| NaHCO_3_ |  |  |  |  | 25 | 100 | 4.17 |  |  |  |
| Tris-Cl |  |  | 10 |  |  |  |  | 10 |  |  |
| Glucose |  | 1 |  | 0.15 | 200 |  | 5.56 |  |  |  |
| Sucrose |  |  | 110 |  |  | 125 |  |  |  |  |
| BSA | *(%)* | 1 | 1 | 1 | 1 | 1 | 1 | 1 |  |  |
| pH |  | 7.6 | 8.3 | 7.6 | 7.9 | 8.2 | 7.6 | 8.4 | 8.3 | 6.5 |
| Osmolarity | *mOsm* | 310 | 300 | 360 | 370 | 305 | 285 | 420 | 1000.9 | 308 |

Kime and Tveiten (KT); TS-2; Ocean pout (OP); Modified turbot (MT); Modified halibut (MH); Hank’s balanced salt solution (HBSS); Smith and Ryan (SR); Sea water (SW).
