# Supplementary material for: Unravelling spermatogenesis in spotted wolffish: Insights from the ultrastructure of juvenile male testes to the cryopreservation of broodstock sperm
Source: Aquaculture. 2024 Nov 15;592:741214. doi: 10.1016/j.aquaculture.2024.741214 (PMC11336258; doi:10.1016/j.aquaculture.2024.741214)
Supplement: Supplementary file 2 — Supplementary material: Supplemental Figure 1. Sperm/extender (1:1). [file mmc2.docx]

**Supplemental Figure 1**

**
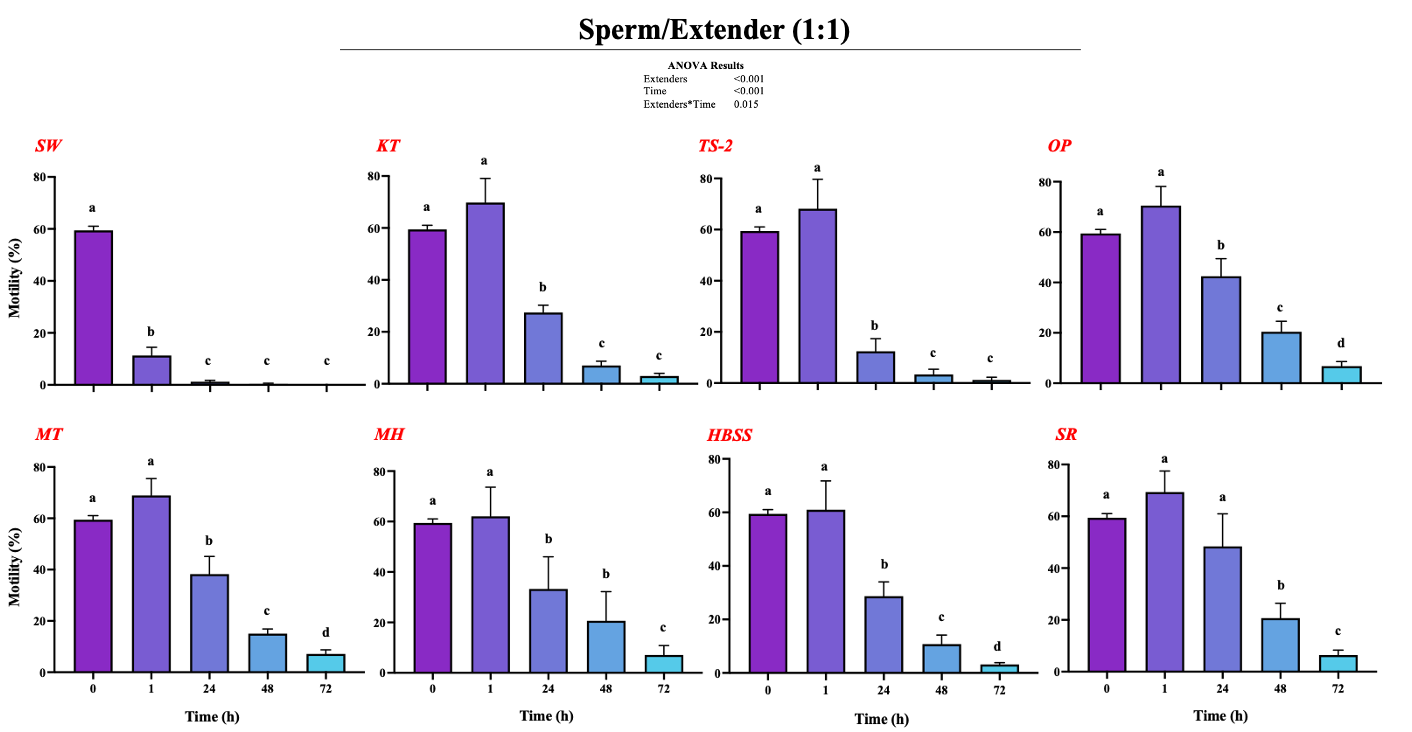
**

**Supplemental Figure 2**. **Effect of extender solutions on CASA parameters of diluted sperm from spotted wolffish broodstock over a 72 hour period under refrigeration at 2-4°C**. Sperm diluted 1:1 with various extenders (SW, KT, TS-2, OP, MT, HBSS, or SR). The parameter evaluated was the motility rate (%). T0 represents the state of fresh sperm without the addition of extender. Statistical analysis was performed by two-way ANOVA (Tukey’s HSD, P ≤ 0.05).
