# Supplementary material for: Unravelling spermatogenesis in spotted wolffish: Insights from the ultrastructure of juvenile male testes to the cryopreservation of broodstock sperm
Source: Aquaculture. 2024 Nov 15;592:741214. doi: 10.1016/j.aquaculture.2024.741214 (PMC11336258; doi:10.1016/j.aquaculture.2024.741214)
Supplement: Supplementary file 3 — Supplementary material: Supplemental Figure 2. Impact of MT and HBSS. [file mmc3.docx]

**Supplemental Figure 2**

**Supplemental Figure 2**. Impact of MT and HBSS on the motility of diluted sperm of spotted wolffish after 72 hours under refrigeration at 2-4°C. The figure supports the notion that no significant difference was observed between MT and HBSS at any time during the 72-hour period. Statistical analysis was performed by one-way ANOVA (Tukey’s HSD test, P ≤ 0.05).
