# Supplementary material for: Unravelling spermatogenesis in spotted wolffish: Insights from the ultrastructure of juvenile male testes to the cryopreservation of broodstock sperm
Source: Aquaculture. 2024 Nov 15;592:741214. doi: 10.1016/j.aquaculture.2024.741214 (PMC11336258; doi:10.1016/j.aquaculture.2024.741214)
Supplement: Supplementary file 4 — Supplementary material: Supplemental Figure 3. Impact of cryoprotectants. [file mmc4.docx]

**Supplemental Figure 3**

**Supplemental Figure 3. Impact of cryoprotectants on CASA parameters of diluted sperm from spotted wolffish broodstock after cryopreservation**. Fresh sperm, diluted at a ratio of 1:3 with various extenders (KT, OP, MT, HBSS or SR), were supplemented with either (**A**) 10% DMSO or (**B**) 10% Methanol (MeOH), and subsequently cryopreserved and maintained in liquid nitrogen. Samples were then subjected to analysis for motility rate (%), curvilinear velocity (VCL; μm/s), straight-line velocity (VSL; μm/s) and average path velocity (VAP; μm/s) at different time intervals over a 24-hour period post-thawing. T0 represents the state of fresh sperm before the cryopreservation procedure. Statistical analysis was performed by two-way ANOVA (Tukey’s HSD, P ≤ 0.05).
